# Sponge spicules as blueprints for the biofabrication of inorganic–organic composites and biomaterials

**DOI:** 10.1007/s00253-009-2014-8

**Published:** 2009-06-01

**Authors:** Werner E. G. Müller, Xiaohong Wang, Fu-Zhai Cui, Klaus Peter Jochum, Wolfgang Tremel, Joachim Bill, Heinz C. Schröder, Filipe Natalio, Ute Schloßmacher, Matthias Wiens

**Affiliations:** 1 Department for Applied Molecular Biology, Institute for Physiological Chemistry, Johannes Gutenberg University, Duesbergweg 6, 55099 Mainz, Germany; 2 National Research Center for Geoanalysis, 26 Baiwanzhuang Dajie, 100037 Beijing, China; 3 Department of Materials Science and Engineering, State Key laboratory of New Ceramics and Fine Processing, Tsinghua University, 100084 Beijing, China; 4 Max Planck Institute for Chemistry, J.J. Becherweg 27, 55128 Mainz, Germany; 5 Institute for Inorganic and Analytical Chemistry, Johannes Gutenberg University, Duesbergweg 10-14, 55099 Mainz, Germany; 6 Materials Synthesis and Microstructure Design, Max-Planck-Institute for Metals Research, Heisenbergstr. 3, 70569, Stuttgart, Germany

**Keywords:** Biosilica, Silicatein, Silintaphin-1, Biomaterials, Porifera, Sponges, Biomedicine, Biotechnology

## Abstract

While most forms of multicellular life have developed a calcium-based skeleton, a few specialized organisms complement their body plan with silica. However, of all recent animals, only sponges (phylum Porifera) are able to polymerize silica enzymatically mediated in order to generate massive siliceous skeletal elements (spicules) during a unique reaction, at ambient temperature and pressure. During this biomineralization process (i.e., biosilicification) hydrated, amorphous silica is deposited within highly specialized sponge cells, ultimately resulting in structures that range in size from micrometers to meters. Spicules lend structural stability to the sponge body, deter predators, and transmit light similar to optic fibers. This peculiar phenomenon has been comprehensively studied in recent years and in several approaches, the molecular background was explored to create tools that might be employed for novel bioinspired biotechnological and biomedical applications. Thus, it was discovered that spiculogenesis is mediated by the enzyme silicatein and starts intracellularly. The resulting silica nanoparticles fuse and subsequently form concentric lamellar layers around a central protein filament, consisting of silicatein and the scaffold protein silintaphin-1. Once the growing spicule is extruded into the extracellular space, it obtains final size and shape. Again, this process is mediated by silicatein and silintaphin-1, in combination with other molecules such as galectin and collagen. The molecular toolbox generated so far allows the fabrication of novel micro- and nanostructured composites, contributing to the economical and sustainable synthesis of biomaterials with unique characteristics. In this context, first bioinspired approaches implement recombinant silicatein and silintaphin-1 for applications in the field of biomedicine (biosilica-mediated regeneration of tooth and bone defects) or micro-optics (in vitro synthesis of light waveguides) with promising results.

## Introduction

Sponges are aquatic, sessile, multicellular organisms with a Bauplan that appears simple at a first glance and lacks similarities to any other living organism. Therefore, during early studies, it was difficult to determine morphological characters that would conclusively allow to group sponges into either one of two kingdoms of multicellular life: Metazoa or Plantae. In an early attempt to reconcile different views, sponges had been classified as “Zoophyta” (Donati 1753) or “Thierpflanzen” (Pallas 1787). Later on, the discovery of significant morphological similarities on the cellular level, i.e., between a highly differentiated poriferan cell type (choanocytes) and unicellular flagellate eukaryotes (choanoflagellates), established a close relationship between the phyla Porifera and Choanozoa (Afzelius 1961; Salvini-Plawen 1978). Recently, phylogenomic analyses also confirmed a significant evolutionary relatedness to the Placozoa. This phylum consists of only one species, which is even simpler in structure than any poriferan species (Blackstone 2009). However, whether Placozoa are highly simplified eumetazoans or a sister taxon to all other metazoans remains controversial until today. It was Grant who first grouped sponges into a common taxon, termed “phylum Porifera” (Grant 1833), initially comprising only sessile marine animals with a soft and spongy (amorphously shaped) body. However, with the discovery of glass sponges (class Hexactinellida; Schmidt 1870), this definition was broadened to include “most strongly individualized, radially symmetrical” entities (Hyman 1940). Finally, after comprehensive isolation, cloning, and phylogenetic analyses of many poriferan genes, it became obvious that the phylum Porifera comprises three classes—Hexactinellida, Demospongiae, and Calcarea—and forms the basis of the metazoan kingdom (Müller 1995). A few years later, it could be clarified that Hexactinellida (glass sponges), Demospongiae (silicate/spongin sponges), and Calcarea (calcareous sponges) are monophyletic and closely related to the common ancestor of all metazoans, the Urmetazoa (Müller 2001).

Sponges appeared during the Neoproterozoic, the geologic period from 1,000 to 542 Ma (reviewed in Müller et al. 2007c). Fossil records indicate that during this period, also other multicellular animals existed, which, however, became extinct (Knoll and Carroll 1999), especially during the Varanger–Marinoan ice ages (605 to 585 Ma). Two major reasons contributed to the evolutionary success of the poriferan taxon: (a) symbiosis with microorganisms and (b) presence of hard skeletons (Müller et al. 2007c). The maintenance of symbiotic relationships with unicellular organisms allowed sponges to survive adverse environmental conditions because the autotrophic microbial symbionts represented rich organic carbon sources. On the other hand, the development of skeletal elements facilitated an increase in size, a common metazoan phyletic trend also known as Cope’s rule (Nicol 1966): Since changes in body size affect almost every aspect of life (Schmidt-Nielsen 1984), two strategies have been developed in animals to circumvent any constraints (reviewed in Page 2007), first by acquisition of a hydrostatic skeleton, as it is known from the “worm”-like phyla of the Ediacara and pre-Ediacara Eon (Xiao and Kaufman 2006), or second by acquisition of rigid solid skeletal elements (Alexander et al. 1979; Biewener 2005), as they were realized in Neoproterozoic siliceous sponges (see Müller et al. 2007c).

**Fig. 1:**
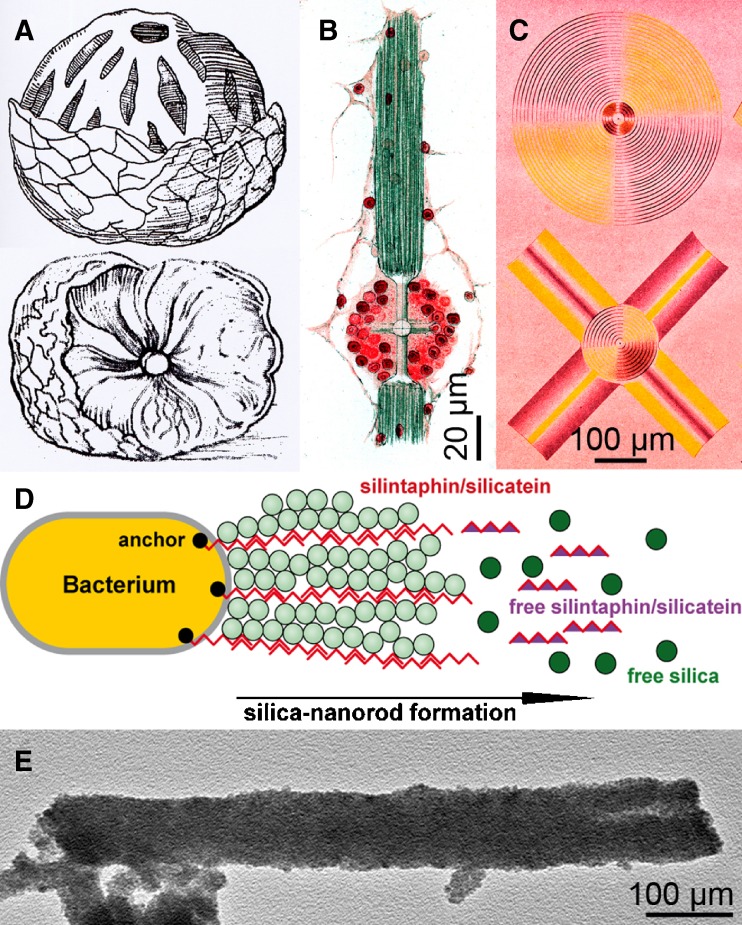
Early illustrations of sponges and their spicules and the modern nanobiotechnological strategies the biological models raised. **A** Oldest illustration of a sponge, *T. lyncurium*, displaying the radially arranged spicule bundles projecting from medulla to cortex (Gesner 1558). **B** The diverse morphology of spicules surrounding a canal system within the hexactinellid *E. marshalli* (Iijma 1901). **C** Refraction of polarized light by spicules from the hexactinellid *M. chuni* (Schulze 1904). Spectral light pattern of a cross-sectioned giant basal spicule (*above*) and a stauractine spicule (*below*) after illumination with two crossed Nicol prisms. **D** Proposed formation of light waveguiding biosiliceous nanorods by bacteria, expressing recombinant silicatein and silintaphin-1 proteins. The scaffold protein silintaphin-1 binds and organizes molecules of the enzyme silicatein that, after addition of orthosilicate, produces and assembles siliceous nanoparticles to rods. **E** Nanoscaled Fe_2_O_3_ rods, formed in vitro by the recombinant proteins silintaphin-1 and silicatein

Skeletal elements (spicules) of siliceous sponges, Hexactinellida and Demospongiae, are composed of amorphous opal (SiO_2_ • *n*H_2_O). They already existed in pre-Ediacaran sponges and represent a general and basic morphological character until today (Xiao et al. 2000). It is easily conceivable why the animals integrated silicon instead of calcium as the fundamental element for their inorganic skeleton, since the Neoproterozoic oceans were rich in silicic acid and continuously replenished by products of the silicate weathering-carbonate precipitation cycle (Walker 2003). Sponges are provided with a sophisticated aquiferous canal system and hence, their body can be stabilized to some extent by internal hydrostatic pressure, even though a vasculomuscular system does not exist. The presence of a siliceous skeleton, however, allowed them to obtain sizes larger than 20 mm, even up to 2.5 m (Wang et al. 2009). Already with the beginning of illustrative descriptions of sponges in 1558 (Gesner 1558), the morphological, physical, and later also the chemical characteristics of spicules were analyzed. The first illustration of a sponge, *Tethya lyncurium*, was given by Gesner (1558; Fig. 1A). It shows bundles of spicules that radiate from the center of the body (medulla) to its surface (cortex). Because of their esthetic beauty especially the morphology of highly filigree, hexactinellid spicules were intensively studied and illustrated. Thus, the pictorial illustrations by Iijma (1901) meticulously depict the architecture of various spicules [different forms of dermalia, pleuralia, comitalia, marginalia, and principalia], with the main bundles protruding upward [prostalia marginalia] and downward [prostalia basalia] of the hexactinellid *Euplectella marshalli* (Fig. 1B). As has often been the case in history, esthetic aspects spawned scientific research and as early as 1904, Schulze published the light refraction pattern of a giant basal spicule from the hexactinellid *Monorhaphis chuni*, generated by polarized light that was obtained by two crossed Nicol prisms (Fig. 1C). These studies allowed the identification of the isotropic properties of siliceous spicules. The chemical characterization of siliceous spicules from the freshwater sponge *Spongia officinalis* had already been described by Gray (1825), outlining their consistency of “pure silica and a little animal matter”. As early as 1904, Schulze discovered that spicules also contain an organic scaffold, a finding that provides the basis for the present day fabrication of inorganic–organic hybrid materials with defined structures (Morse 1999; Wiens et al. 2009; Fig. 1D, E). This achievement became possible since the genetic toolbox for the synthesis of spicules had been decoded, comprising two proteins, silicatein as the basis for the formation of the inorganic matrix (Cha et al. 1999; Krasko et al. 2000) and silintaphin-1 as the structure-providing scaffold (Wiens et al. 2009). It is the aim of this review to summarize the role of both molecules during spicule formation and to highlight their application for the biofabrication of new nanoscale hybrid materials and composites.

## Biomineralization

During animal evolution, biomolecules (e.g., secondary metabolites) and biomaterials (e.g., biominerals) were selected for higher biological efficiency and superior physical properties. Scheuer (1987) and Baker and Murphy (1981) were pioneers in exploiting secondary metabolites for biomedical applications. They conclusively outlined strategies to discover and apply such compounds for biomedical use, resulting in the development of 9-β-d-arabinofuranosyladenosine (ara-A) as a first active pharmaceutical ingredient (Müller et al. 1977). On the other hand, the fundament for the biotechnological exploitation of biominerals has been laid by Lowenstam and Weiner (1989), who introduced the conceptual framework for understanding the formation of inorganic deposits within organisms. In this context, the fundamental importance of organic macromolecules during biomineralization was highlighted.

**Fig. 2:**
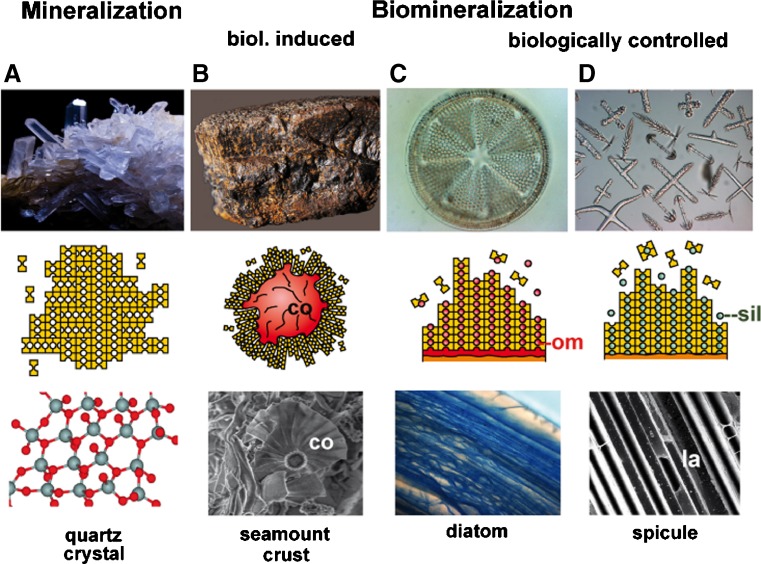
Mineralization versus biomineralization. **A** Mineralization process: example, quartz crystal formation. Inorganic monomers of silicic acid form crystals with defined chemical compositions and physical structures in a hydrothermal environment and under high pressure. **B** Biologically induced mineralization: example, ferromanganese crust formation in the deep sea. Coccospheres (*co*) of biogenic origin serve as organic template for mineral deposition. **C** Biologically controlled mineralization: example, frustule formation in the diatom *Cyclotella antigua*. Bioseeds and organic matrices (organic guiding macromolecule (*om*)) control initiation and growth of the biomineral. **D** Unique form of biologically controlled mineralization: example, spicule formation in the hexactinellid *Hyalonema mirabile*. Spicules are formed by the enzyme silicatein (*sil*) and the scaffold protein silintaphin-1, using a soluble silicon source. Both molecules also guide the assembly of siliceous nanoparticles to concentric silica lamellae (*la*)

During inorganic mineralization, the conversion of monomers (e.g., metals or their salts) into solid-state material usually occurs through endothermic reactions. The products are characterized by a defined chemical composition/physical structure and can be amorphous or crystalline. Quartz crystals [SiO_2_] for example are formed in watery solution under hot conditions, in hydrothermal environments, and often at very high pressures. Since in the aqueous milieu hardly any free SiO_2_ molecules are available, crystal growth proceeds by progressive and layered deposition of dissolved orthosilicic acid [H_4_SiO_4_] at the surface of an inorganic solid seed (Fig. 2A).

In contrast, for initiation and maintenance of biomineral formation, bioseeds and/or organic surfaces and matrices are required; for these reactions, inorganic [e.g., phosphate] or organic precursors [β-glycerophosphate] are used as donors. Weiner and Dove (2003) distinguished two categories of mineralization: (a) biologically induced mineralization and (b) biologically controlled mineralization. (a) During the seed phase of biologically induced mineralization processes, organic polymers allow controlled nucleation and crystal growth. For example, marine snow (three-dimensional organic/mineral meshwork; Leppard 1999) and coccoliths/coccospheres (calcitic remains of coccolithophorids [single-celled algae]) have mineralization activity. The latter one has recently been implied in the formation of ferromanganese crusts in the deep sea (Wang and Müller 2009a, b; Fig. 2B). These particles/aggregates act as bioseeds and mediate deposition of inorganic materials from an environment that contains the inorganic precursors at nonsaturated conditions. (b) Biologically controlled mineralization describes a process that is guided along bioseeds and organic matrices (organic guiding macromolecules; Fig. 2C). These biomolecules control initiation and growth of biominerals, morphology, and speed of the mineralization process. Biominerals represent genuine composite materials, formed from the inorganic “polymer”/mineral and the organic component (protein, polysaccharide, glycoprotein). These organic molecules function as bioseeds (during seed phase) and also as scaffold during the subsequent growth phase. This widely occurring process takes place within the living system (e.g., mammalian teeth or bone formation) or extracellularly (e.g., foraminiferan CaCO_3_ shells; see Lowenstam and Weiner 1989).

A special form of biologically controlled mineralization (i.e., enzymatically controlled; Fig. 2D) has been described for the biosilicification process in siliceous sponges (see Müller et al. 2007d). In these animals (Demospongiae and Hexactinellida), the enzyme silicatein is catalytically involved in the formation of biosilica (Cha et al. 1999; Müller et al. 2005; 2007d), concurrently serving as organic scaffold for the inorganic polysilicate mineral product (Müller et al. 2008a; Wang et al. 2008). Hence, it acts as both bioseed and organic matrix. Since then, learning from sponges and mastering nature’s concept of forming siliceous skeletal elements inspired many strategies that aim to biofabricate minerals with exquisite and distinguished (bio)chemical and (bio)physical properties (Mayer 2005). Additionally, the discovery of organic molecules that are implicated in the formation of inorganic polymers caused a paradigm shift in biomaterials science and technology. While silicatein might facilitate the synthesis of new biomaterials (e.g., for dental applications or tissue engineering), an additional protein, silintaphin-1, provides the products of the silicatein-mediated reactions with defined morphologies.

## Silicatein-based spicule formation

The biogenic basis of spicule formation and the turnover of silica in spicules have already been depicted by Duncan (1881). He formulated “The spicule which has lived, has to decay, and may live again in another form”. However, it took until 1999 until Cha et al. discovered that the main constituent of the proteinaceous filament within the axial canal of spicules is an enzyme, subsequently termed silicatein, which might be involved in biosilica formation. Soon after having identified this anabolic enzyme, also the corresponding catabolic enzyme (silicase) was discovered (Schröder et al. 2003). The identification of a biosilica degrading enzyme supported the view that the siliceous components in spicules are under metabolic turnover (Eckert et al. 2006). Studies on the metabolism of spicules on the cellular level became possible after the introduction of a poriferan cell culture system, primmorphs (Imsiecke et al. 1995; Custódio et al. 1998). Already the first contribution on that topic resolved that spicule formation starts intracellularly in “J” sclerocytes, by formation of an initial organic axial filament, around which the inorganic silica mantel is deposited. This result had later been corroborated by application of more advanced immunochemical and electron microscopy techniques (Müller et al. 2007e).

### The enzyme

**Fig. 3:**
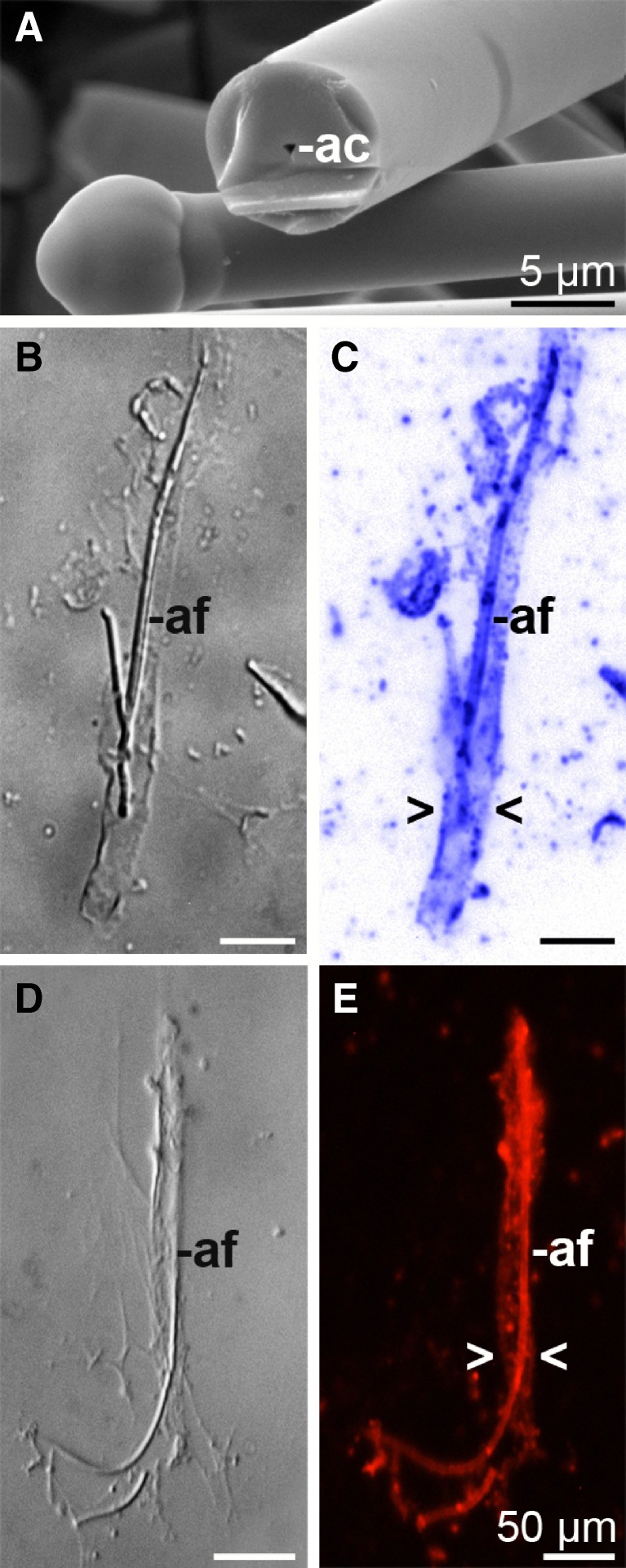
Protein components within poriferan siliceous spicules. **A** Broken *S. domuncula* (Demospongiae) tylostyle, displaying the axial canal (*ac*), which harbors the proteinaceous axial filament. Tylostyles are uniradiate spicules displaying one pointed end and a knob at the other; high resolution scanning electron microscopy. **B**, **C** Dissolution of a spicule via HF vapor, releasing both proteinaceous axial filament (*af*) and coating (*> <*) that can be stained by Coomassie brilliant blue. **D**, **E** Immunodetection of silicatein within axial filament (*af*) and coat (*> <*)*,* using fluorescently labeled antibodies

Thorough dissolution of the inorganic silica from spicules (Fig. 3A; *Suberites domuncula* [demosponge] spicules) with hydrofluoric acid (HF) vapor revealed in addition to the presence of the axial filament a spicule-enfolding proteinaceous coat. This coat can be stained with Coomassie brilliant blue (Fig. 3B, C) and more specifically with fluorescently labeled antibodies against silicatein (Fig. 3D, E). After the discovery of the cathepsin L (cysteine protease)-related silicatein (Shimizu et al. 1998; Cha et al. 1999) in spicules of the demosponge *Tethya aurantium*, several related genes were elucidated in both marine and freshwater demosponges (reviewed in Müller et al. 2007e). The corresponding deduced polypeptides comprise about 325 amino acids (aa) with a molecular weight of ca. 35 kDa. During maturation, this primary translation product (proenzyme) is processed by cleaving off a signal peptide (aa_1_ to aa_17_; *S. domuncula* [demosponge] silicatein-α) and the adjacent propeptide (aa_18_ to aa_112_), resulting in the mature enzyme that has a size of 24–25 kDa. Similar to cathepsins, the catalytic center of silicatein contains His and Asn. However, the Cys of the cathepsins’ catalytic triad is exchanged by Ser in silicatein. In addition to about ten putative protein kinase phosphorylation sites, silicateins display a cluster of serine residues that is found close to the central aa residue of the catalytic triad, but is otherwise missing in cathepsins. Subsequent phylogenetic analyses revealed that silicateins form a separate branch from cathepsins (Müller et al. 2007e).

The difficult accessibility of hexactinellids, which live primarily in depths of more than 300 m, generally results in a very poor sampling. Accordingly, only recently the first hexactinellid silicatein (*Crateromorpha meyeri*) could be identified and characterized (Müller et al. 2008c). This molecule shares high similarity to the demosponge sequences (expect value of 8e^−58^) and contains the same catalytic triad amino acids. However, striking in the *C. meyeri* sequence is a second Ser-rich cluster, which is located between the second and the third aa of the catalytic triad. Strong binding of the protein to the spicule silica surface has been attributed to this cluster (Müller et al. 2008a).

The posttranslational modifications of silicatein have been found to be essential for the enzyme activity with respect to (a) association with other structural and functional molecules within the tissue and (b) self-association/self-assembly. For those studies, silicatein had been isolated from spicules in the absence of HF, but in the presence of a glycerol-based buffer. Following this rationale, it could be demonstrated that silicatein exists not only in the axial canal but also in the extraspicular and extracellular space (Müller et al. 2005; Schröder et al. 2006). The enzymatic reaction mechanism of silicatein had been proposed by Cha et al. (1999); the detailed properties of the reaction kinetics have been specified experimentally (Müller et al. 2008b).

### Spiculogenesis

**Fig. 4:**
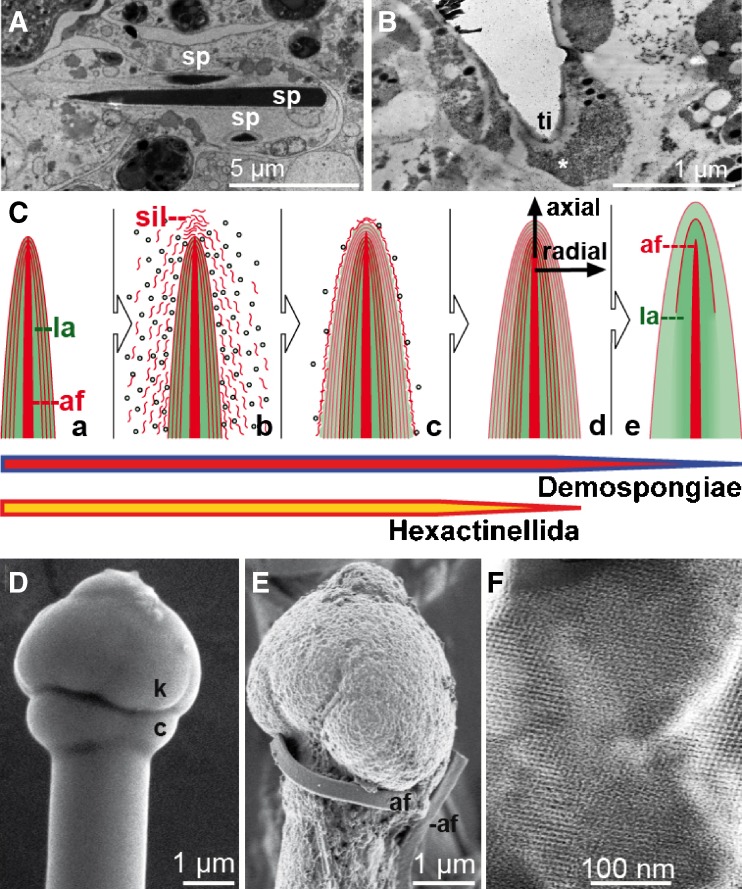
Formation of poriferan siliceous spicules. **A** Initial intracellular stage in sclerocytes. Three spicules (*sp*; *S. domuncula*) are formed within silicasomes in the same sclerocyte, HR-SEM. **B** Longitudinal section of an intracellular spicule. Immunocomplexes of silicatein antibodies/nanogold-labeled secondary antibodies (*asterisk*) appear accumulated at the tip (*ti*) of the growing spicule. **C** Schematic outline of radial/axial growth and maturation of spicules during the extracellular stage of spiculogenesis. *a* After extrusion of a spicule that is composed of one or only a few lamella(e) (*la*) into the extracellularly space an enfolding organic cylinder is formed. *b*, *c* This cylinder consists of silicatein molecules, beaded along strings of galectin. Within the cylinder, siliceous layers are formed consecutively. These siliceous lamellae mostly remain distinct (*d*; Hexactinellida) or fuse completely by a biosintering process (*e*; Demospongiae), creating a “solid” siliceous mantel that surrounds the axial filament (*af*). **D** The tylostyle head, the knob (*k*), is based on a collar (*c*), until the spicule elongates and forms the monaxonal pin (*S. domuncula*); HR-SEM. **E** After exposure of the tylostyle to HF vapor, the silica is progressively dissolved, exposing granular structures. Finally, the axial filament (*af*) is released that has been entangled around the knob. **F** Crystal*-*like pattern within the amorphous opal of the spicules from the hexactinellid *A. ramosus*
- *Extracellular phase (consecutive appositional growth)*: Silicatein is present in an enzymatically active form in the extracellular space (Müller et al. 2005). There, silicatein molecules are organized to larger entities as demonstrated by immunogold electron microscopic analysis. These molecules are arranged along filamentous strings, which are organized concentrically to the spicule surface (Schröder et al. 2006; Fig. 4C) and consist of the protein galectin that oligomerizes in the presence of Ca^2+^. Within this organic cylinder that enfolds the growing spicule, the siliceous mantel grows stepwise, by appositional layering of lamellae. Not only centrifugal growth (“thickening”) but also axial growth (“elongation”) of spicules is driven by extraspicular silicatein. Thus, the accumulation of silicatein at the tip of growing spicules can be visualized immunochemically by transmission electron microscopy (TEM) analyses (Fig. 4B). In the extracellular space, both axial and radial growth of the spicules is driven by silicatein that surrounds the surface of the already existing silica lamellae (Fig. 4C-a–C-d). In hexactinellids, appositionally layered silica lamellae can reach 1,000 in number (Wang et al. 2009). However, in demosponges, the individual lamellae fuse and form a “solid” siliceous shell, which surrounds the axial filament (Fig. 4C-e).
- *Extracellular phase (final morphogenesis)*: So far, the processes described above do not explain the species-specific shaping of spicules. This final step of spiculogenesis (i.e., morphogenesis) still remains mysterious. However, since spicules of both demosponges (e.g., *S. domuncula*, Eckert et al. 2006) and hexactinellids (e.g., *M. chuni*, Müller et al. 2008d) are surrounded or even embedded into a fibrous network of collagen and other proteins (see below), it is safe to assume that these molecules (released by specialized sponge cells) provide a scaffold within which the galectin-containing strings are organized (Fig. 5). Data suggest that the galectin-containing strings are organized by collagen fibers to net-like structures (Schröder et al. 2006). Those fibers that are released by the specialized cells, the collencytes, provide the organized platform for the morphogenesis of the spicules. The longitudinal growth of the spicules can be explained by the assumption that at the tips of the spicules, the galectin/silicatein complexes are incorporated into deposited biosilica under formation and elongation of the axial canal.

The process of spicule formation can be divided into an initial intracellular step and a subsequent extracellular shaping phase:

- *Intracellular phase (initial growth)*: It could be demonstrated that silicic acid is actively taken up by cells [sclerocytes] via the Na^+^/HCO_3_^−^[Si(OH)_4_] cotransporter (Schröder et al. 2004). In parallel, mature silicatein is synthesized/processed and subsequently deposited together with silicic acid in special organelles of the sclerocytes, the silicasomes. Within silicasomes axial filaments are formed around which silica is subsequently deposited enzymatically (Fig. 4A, B). After formation of a first layer (or a few layers), juvenile spicules are released into the extracellular space, where they grow in length and diameter by appositional layering of silica lamellae (Müller et al. 2005; Fig. 4C). There, spicules obtain their final shape, e.g., *S. domuncula* tylostyles are characterized by a terminal globular swelling (Fig. 4D, E).

**Fig. 5:**
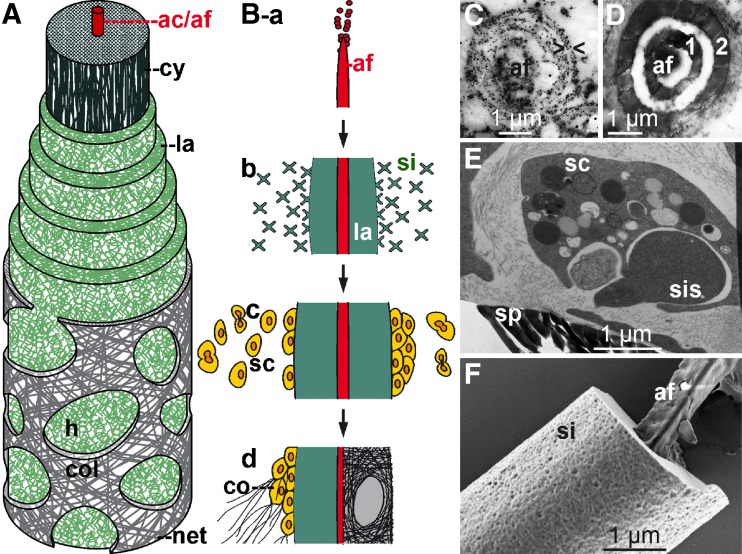
Control mechanisms involved in the morphogenesis of siliceous spicules. **A** Schematic representation of the three morphological zones of spicules (the giant basal spicule of *M. chuni* has been used as model), comprising the axial canal (*ac*; axial filament (*af*)), the axial cylinder (*cy*; axial barrel) and the lamellar zone (*la*; lamellar coating). The spicule surface is surrounded by a proteinaceous coat (*net*) that contains collagen (*col*), leaving open pores (*h*; <10 μm), which allow access to the biosilica lamellae. **B** Biosilica synthesis, assembly, and morphogenetic effects. *a* At first, the axial filament (*af*) is formed intracellularly. *b* Silicatein/silintaphin-1 within the filament mediate the formation of biosilica and its subsequent assembly to lamellae. *c* Biosilica in the lamellae attracts undifferentiated cells (stem-cell like) and subsequently *d* induces cell differentiation into sclerocytes (biosilica-forming cells, *sc*) and collencytes (collagen-forming cells, *co*). These two cell types facilitate the synthesis of a fibrous proteinaceous net (*net*), surrounding the spicules. **C** Immunogold labeling of silicatein to visualize the concentric arrangement of the enzyme (*> <*) around a center with granules of a higher density, highlighting the prospective axial filament (*af*); TEM. **D** Fracture pattern of a juvenile spicule, giving rise to two distinct concentric siliceous layers, operationally termed lamella-1 (*1*) and lamella-2 (*2*). In the center canal, the axial filament is seen; TEM. **E** Release of electrodense material (possibly silicon-related) from sclerocytes (*sc*) adjacently localized to spicules (*sp*) into the extracellular space. The organelles that contain electrodense granules were termed silicasomes (*sis*); TEM. **F** Mature siliceous spicule (*si*) from *S. domuncula* displaying the axial filament (*af*) that protrudes from the fracture site; HR-SEM

### Silica deposition in spicules

A comprehensive summary of the physical and chemical analyses of poriferan siliceous spicules has been given by Sandford (2003). It is summarized that in both classes of siliceous sponges, the inorganic matrix is almost indistinguishable and comprises amorphous opal, which contains traces of other elements. Weaver et al. (2003) conclusively demonstrated that in spicules of the demosponge *T. aurantium*, silica nanoparticles, with a mean diameter of 75 nm, are arranged along alternating layers. This pattern had been exposed after etching of spicular cross sections. It was concluded that the annular layering may be related to variations in the degree of silica condensation, rather than variability in the inclusion of organics. Nevertheless, a proteinaceous coat, immunoreacting with silicatein antibodies, can be released by HF treatment from spicules *S. domuncula* (Fig. 3). Similarly, the exact localization of proteins within the lamellar organization of hexactinellid spicule remains to be determined. While Woesz et al. (2006) provided evidence by Raman spectroscopy for the existence of proteins between siliceous layers (organic interlayers in *M. chuni*), high resolution scanning electron microscopy (HR-SEM) and biochemical determinations suggest that proteins are localized within the silica depositions (Müller et al. 2008d). Nonetheless, the unusual combination of mechanical properties, such as strength, stiffness, and toughness observed in hexactinellid spicules, is based on the proteinaceous components enfolded within (Mayer 2005).

**Fig. 6:**
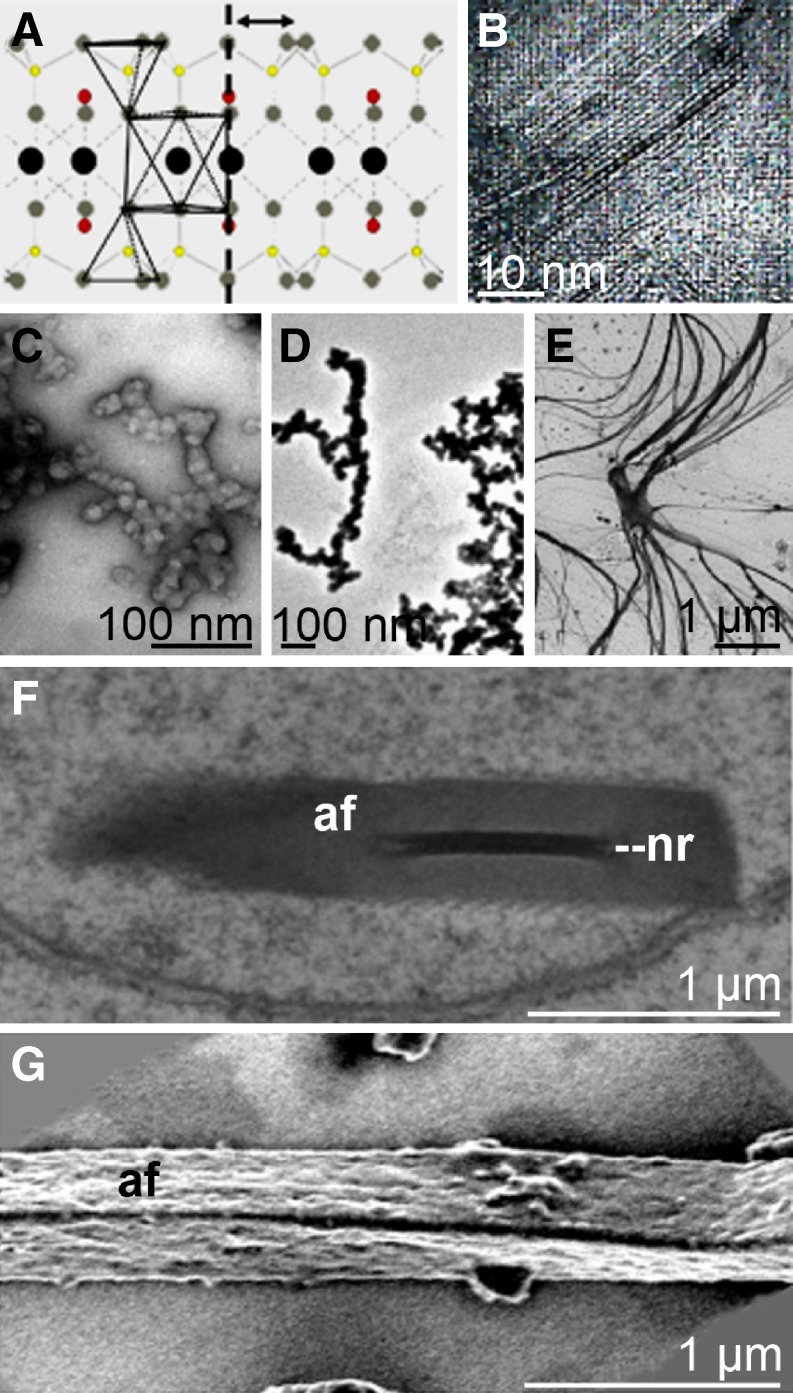
Transient manifestation of crystalline nanorods in the axial filament of siliceous spicules (*S. domuncula*). **A** Scheme of a two-layer smectite. **B** The nanorod crystals show a general layered structure, with a 10-Å distance, arranged parallel to the major growth direction of the rod; HR-TEM. **C**–**E** Oligo-/polymerization of silicatein. Silicatein was prepared from spicules (*S. domuncula*) and was then allowed to reassemble in a glycerol-free buffer. After incubation periods of 30 (**C**), 180 (**D**), or 360 min (**E**), samples were taken and analyzed by TEM. **F** Developing axial filament in silicasomes that are not yet surrounded by a silica lamella. Such filaments frequently contain nanorods (*nr*); TEM. **G** Terminally differentiated axial filament, released from an axial canal of a *S. domuncula* tylostyle

Very recently, first evidence has been presented that demosponge spicules comprise, within the amorphous silica constituent, mesopores with widths and lengths of 23 and 110 nm (Jensen et al. 2009). Identical patterns, reminiscent of crystalline structures, have been discovered in hexactinellid spicules, e.g., of *Aphrocallistes ramosus* (Fig. 4F). The existence of crystals (“intranuclear crystals”) in the demosponges *Ephydatia muelleri* and *Spongilla lacustris* had already been described as early as 1995 (Imsiecke and Müller 1995). Very recently, crystals with a size of 30–500 nm could be visualized by HR-TEM and electron dispersion X-ray spectroscopy (EDX; Mugnaioli et al. 2009). Subsequent EDX with automated diffraction tomography corroborated the presence of smectite crystals (Fig. 6A, B). Interestingly, those crystals are associated with the axial filament where they exist only transiently. It is postulated that the smectite crystals are involved in the initial assembly of silicatein monomers and organization of axial filaments. At present, it is suggested that the crystal formation occurs concurrently to the fractal-like assembly of silicatein. Fractal structures from associating silicatein oligomers have been described for both demosponges (*T. aurantium*, Murr and Morse 2005; *S. domuncula*, Müller et al. 2007a) and hexactinellids (*M. chuni*, Wang et al. 2008). After the initial assembly of monomeric silicatein preparations (Fig. 6C, D), filamentous structures are formed (Fig. 6E). Here, it is hypothesized that the smectite crystals contribute to and facilitate the formation of the primary axial filaments (<500 nm; Fig. 6F), which have not yet started to synthesize silica. In filaments that had been isolated from mature spicules, those nanorods have not been found (Fig. 6G).

## Biosintering

**Fig. 7:**
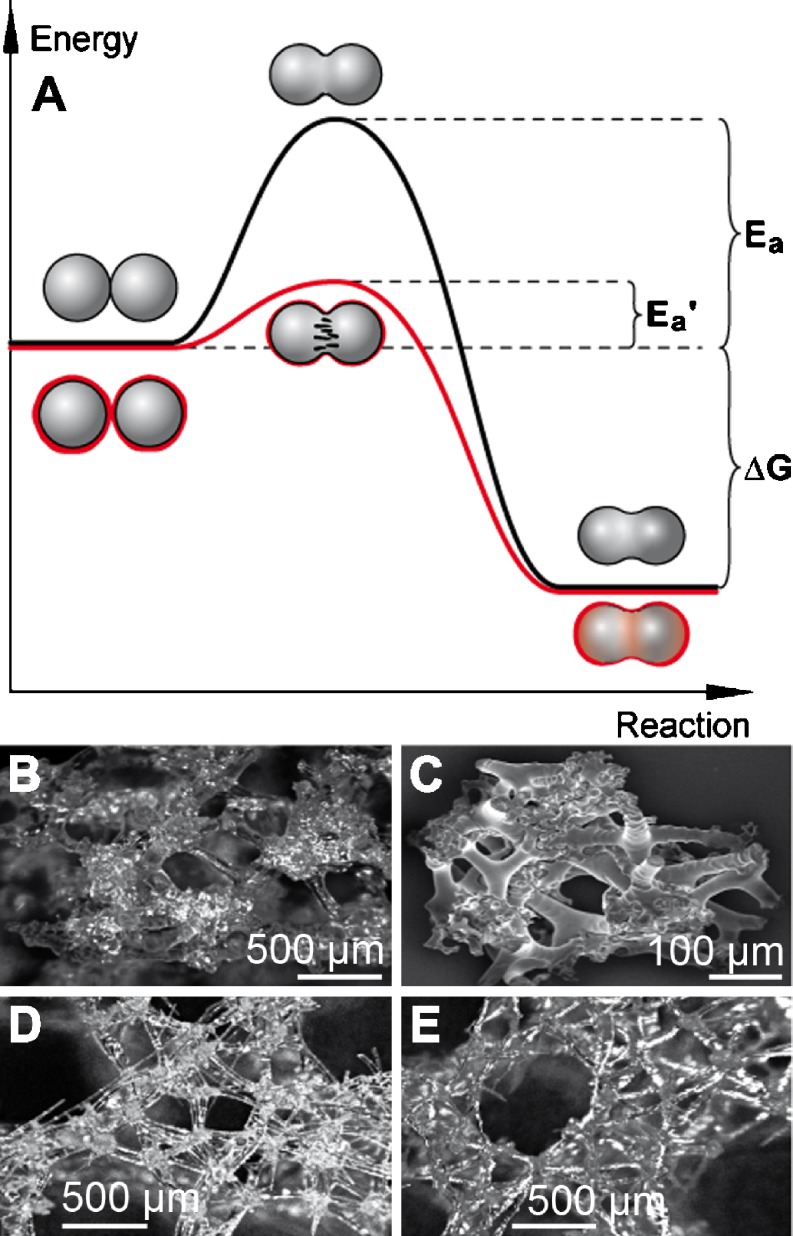
Proposed biosintering mechanism of silica nanospheres within and between siliceous spicules. **a** To initiate conventional sintering processes, activation energy (*E*_a_) is required since fusion of inorganic particles is exergonic (Δ*G* negative). During biosintering, however, the magnitude of *E*_a_ is reduced due to the presence of the enzyme silicatein (*E*_a_′). This reduction facilitates and accelerates the fusion process at ambient temperature and allows the free energy (Δ*G*) for the sintering process to be released. It is outlined that the silica nanoparticles are surrounded by silicatein (in *red*). **b**, **c** Joining but not fusion of the spicules in demosponges, e.g., the lithistid sponge *D. japonica*. It is shown that the megascleres/microscleres (desmas) become hypersilicified and interlock but do not fuse. **d**, **e** Spicules from the hexactinellid *Aphrocallistes vastus* fuse and form biosilica “units”

As outlined above, the basic pattern of silica growth around axial filaments is identical in spicules of demosponges and hexactinellids, with one exception: While in demosponges all silica lamellae fuse to a “solid” structure, a similar process occurs in hexactinellids only in the central part of spicules restricted to Amphidiscosida (e.g., the giant basal spicules from *M. chuni*, Wang et al. 2009; Fig. 4C). In most other hexactinellid taxa, the lamellae remain distinct. In demosponges, the individual lamellae merge by fusion of the 70–300-nm large silica nanospheres the lamellae are composed of (Tahir et al. 2004). Whereas this process occurs in the living organism at ambient temperature, fusion of quartz glass grade by melting processes would require temperatures well above 1,800°C. Accordingly, the product of this biological fusion of silica lamellae resembles the product of a technical process termed sintering, i.e., a thermally activated material transport in a powder or porous compact, decreasing the specific surface by growth of the particle contacts, shrinkage of pore volume, and change of the pore geometry (Thümmler and Oberacker 1993; Wakai and Aldinger 2004). In general, the material is densified below its melting point. Sintering is widely used for the densification of oxide-based ceramic powders including silicon oxide and requires in general temperatures above 1,000°C for thermal activation. The free enthalpy (Gibb’s energy; Δ*G*) of sintering is exergonic, implying that during the reaction energy is released, provided that the activation energy (*E*_a_; reaction minimum energy required to start a chemical reaction) has been overcome. Enzymes work by lowering the activation energy for a reaction and thus dramatically increase the rate of the reaction. Considering the fact that within the silica mantel of spicules the enzyme silicatein exists (either within (Müller et al. 2008a; d) or between (Woesz et al. 2006) the lamellae), silicatein would be a prime candidate to reduce *E*_a_ of this exergonic reaction (Fig. 7A). Consequently, it acts in principle like the sintering additives used in conventional powder technology processes. Therefore, we propose that the fusion of silica lamellae in demosponge spicules follows a newly defined biocatalytically mediated process, “biosintering”. Accordingly, biosintering occurs during formation of poriferan siliceous spicules. Similar to demosponges in hexactinellids, fusion between spicules is frequently observed in the orders Hexactinosida and Lychniscosida (Uriz 2006). There, the initial skeletal elements, composed of hexactine spicules, are subsequently reinforced by additional silica. In Fig. 7D, E, the resulting biosilica “units”, comprising micro- and megascleres, are shown. In contrast, in the taxonomic group of lithistid demosponges (e.g., *Discodermia japonica*), spicules intimately interlock with each other, without fusion (Pisera 2003; Uriz 2006; Fig. 7B, C).

## The silicatein interactor silintaphin-1

**Fig. 8:**
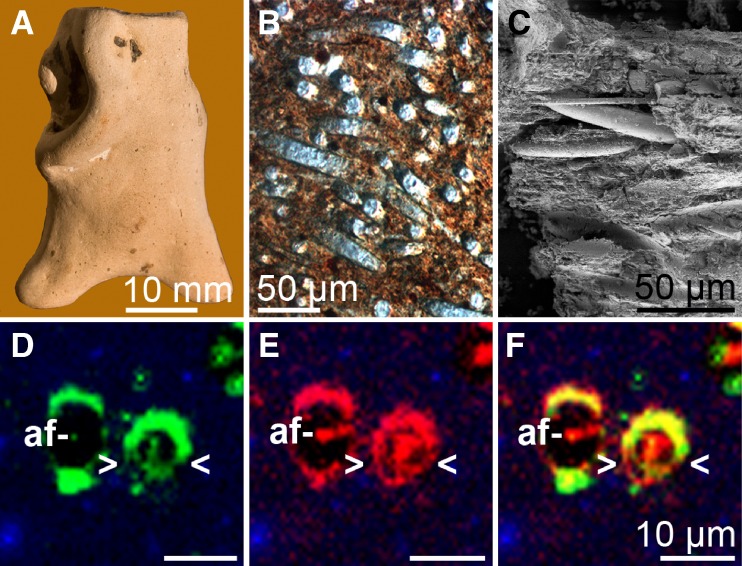
Protein components of siliceous spicules. **A**–**C** Ancient pottery from the Orinoco River region (**A**). The clay had been reinforced by spicules during its production (**B**, **C**). **D**–**F** Colocalization of silintaphin-1 with silicatein in tissue sections from *S. domuncula*. Sections were incubated with antibodies raised against silintaphin-1 and silicatein. Then, immunocomplexes were detected with fluorescently labeled secondary antibodies (Alexa Fluor 488- [**D**, silintaphin-1] or Cy3-labeled [**E**, silicatein]) via confocal laser scanning microscopy. The merged channels revealed a significant colocalization of both proteins (*yellow*) within both the axial filament (*af*) and a massive layer surrounding the spicules (*> <*; **F**)

Silicatein-based synthesis of biosilica composites is of great interest for many industrial applications (communications, microelectronics, micro-optics, etc.; Schröder et al. 2008; Brutchey and Morse 2008). However, the mechanical properties of the synthetic composites (i.e., amorphous nanoparticles) so far do not meet the requirements for such applications, even though the natural model (i.e., spicules) is provided with exceptional strength, stiffness, toughness, and distinct morphology (Mayer 2005). These characteristics already had been exploited in former times in many regions of the world, where spicules were used in ancient ceramics as temper to fiber-reinforce pottery from the Neolithic (McIntosh and MacDonald 1989). Samples from the region around the Orinoco River are shown (Linné 1932; Fig. 8A–C). However, the recent discovery of a so far unknown poriferan protein led to a significant breakthrough that allows regulating morphology and chemical composition of silicatein products.

In order to obtain the molecular tools required to enzymatically generate composite materials with defined characteristics, a comprehensive screening program was started for proteins that mediate in combination with silicatein spiculogenesis. By application of a yeast two-hybrid system, a poriferan (*S. domuncula*) cDNA library was successfully screened for a silicatein-binding protein involved in spiculogenesis. Thus, a first strong silicatein interactor was identified and subsequently termed silintaphin-1 (Wiens et al. 2009). The existence of this 42.5-kDa silicatein interactor (386 aa) is restricted to sponges. Since it shows no sequence similarities to any other known proteins, silintaphin-1 probably represents the prototypic member of a new family. However, the protein reveals significant similarities along a stretch of ca. 110 aa to the pleckstrin homology (PH) domain, a common protein interaction domain that occurs in many unrelated proteins. PH domains usually form a structure that can serve as scaffold for presenting different types of binding sites (Lemmon and Ferguson 2000; Lemmon 2004). In silintaphin-1, the PH domain likely facilitates binding to silicatein and consequently assists assembly of the axial filament. Furthermore, it is flanked on both sides by several nearly identical aa sequence repeats rich in Pro and charged/polar residues (Glu, Asp, Thr). This combination of repeated sequence composition and ligand-binding domain is also found in titin (connectin), a filamentous protein of sarcomers with unique elastic properties and distinct bending rigidities (Trombitas et al. 1998). In analogy, silintaphin-1 hence might convey a similar elasticity and flexibility required during spiculogenesis.

Silintaphin-1 expression was observed to be significantly upregulated in regenerating *S. domuncula* tissue that requires profound synthesis of spicules and/or reorganization of the skeletal elements. Furthermore, the expression was also susceptible to the silicon concentration in the surrounding aquatic medium, indicating that silintaphin-1 is tightly interwoven in the pathways of poriferan silicon metabolism and spiculogenesis.

Subsequently, antibodies were raised against the poriferan protein, to elucidate its localization in sponge tissue. In situ detection of silicatein with labeled antibodies was already performed earlier, confirming the presence of the enzyme in axial filaments (Müller et al. 2005). However, recent analyses by confocal laser scanning microscopy conclusively demonstrated the almost exclusive colocalization of silintaphin-1 (fluorescein isothiocyanate-labeled antibodies; Fig. 8D) and silicatein (Cy3-labeled antibodies; Fig. 8E) within both a substantial layer surrounding spicules and the axial filament (Fig. 8F; merge). Interestingly, within the axial filament silintaphin-1 forms a core structure that is enfolded by silicatein. The general colocalization with silicatein suggests that silintaphin-1 contributes not only to the filament formation but also to the determination of the final spicular morphology.

## Biomedical/biotechnological application of silicatein and silintaphin-1

**Fig. 9:**
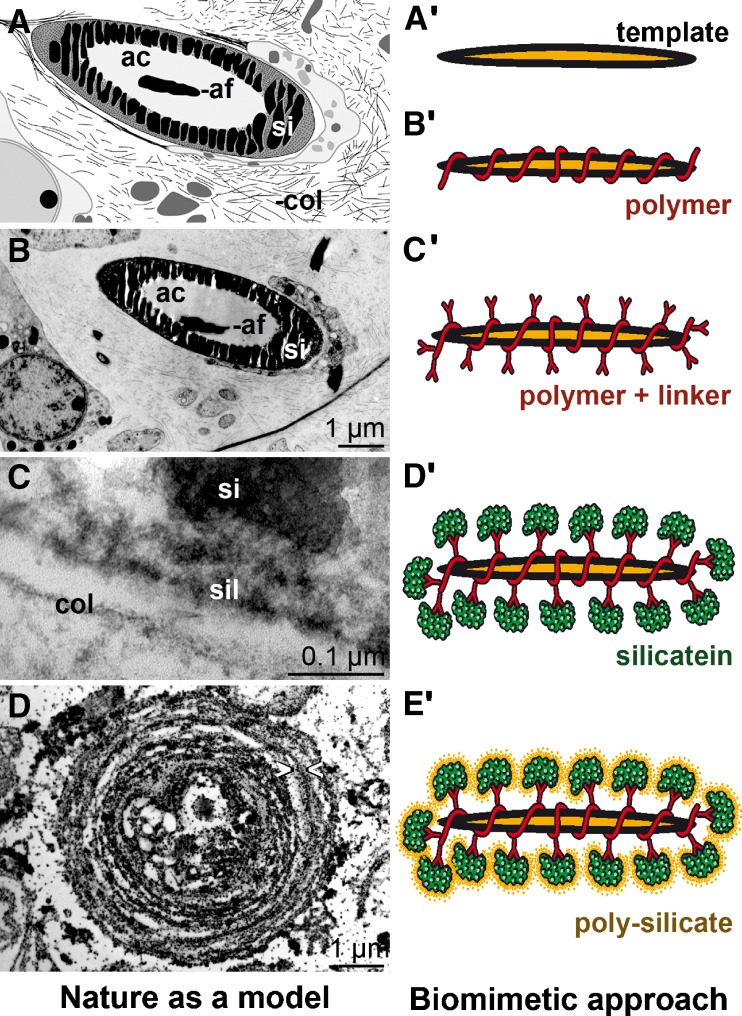
“Nature as model”—a biomimetic approach. *Left panel*: **A**–**D** Synthesis of poriferan spicules in the extracellular space. **A** (scheme) and **B** (TEM) Cross section through a growing spicule with the silica coat (*si*) that surrounds axial canal (*ac*) and axial filament (*af*). In the extracellular space collagen (*col*), fibers are indicated. **C** High magnification TEM image showing the silicatein molecules (*sil*), associated with galectin, surrounding the silica material (*si*). Collagen fibers (*col*) have no contact to the silicatein/silica complex. **D** Immunogold-labeled silicatein visualizing the organic cylinders which guide and mediate the synthesis of the silica layers in an appositional manner (marked *> <*). *Right panel*: **A'**–**E'** The biomimetic approach. The template (**A'**) is successively functionalized with a reactive ester polymer (**B'**) and the NTA linker (**C'**). **D'** Recombinant silicatein is bound via His-tag and Ni^2+^ to the NTA-polymer and subsequently mediates formation and assembly of polysilica formation (**E**′)

After the initial intracellular stage of spiculogenesis, the developing spicule is transported into the extracellular space, where it grows through apposition of lamellar silica layers. There, spicules obtain their final sizes, between 450 μm (Demospongiae) and 2.5 m (Hexactinellida), and morphology. Based on the processes observed during the extracellular growth of spicules, the natural principle was applied during biomimetic and (nano)biotechnological approaches (“Nature as Model”). As mentioned above, growth of spicules is guided by a proteinaceous cylinder containing silicatein that facilitates the formation of biosilica lamellae (Fig. 9; left panel [A–D]). In a bioinspired approach, biosilica was synthesized on “inert” surfaces (matrices) from monomeric precursors (Tahir et al. 2004). However, prior to the biosilica formation, the matrices had to be functionalized with a reactive polymer that is subsequently able to chemisorb nitrilotriacetic acid (NTA), which in turn is required to bind His-tagged recombinant silicatein (Fig. 9; right panel [A′-E′]). Silicatein that had been immobilized onto this matrix has the capacity to synthesize nanoparticulate biosilica, biotitania, and biozirconia from monomeric precursors. This striking example shows that nature could be used as a biological blueprint for biomedical and biotechnological applications. With this concept, a new approach was introduced, the synthesis of inorganic polymers enzymatically mediated by organic molecules. In a complementary approach, Wöhler (1828) succeeded almost 200 years ago with his seminal experiments on the synthesis of urea, an organic compound, from inorganic materials.

### Biomedical approach

**Fig. 10:**
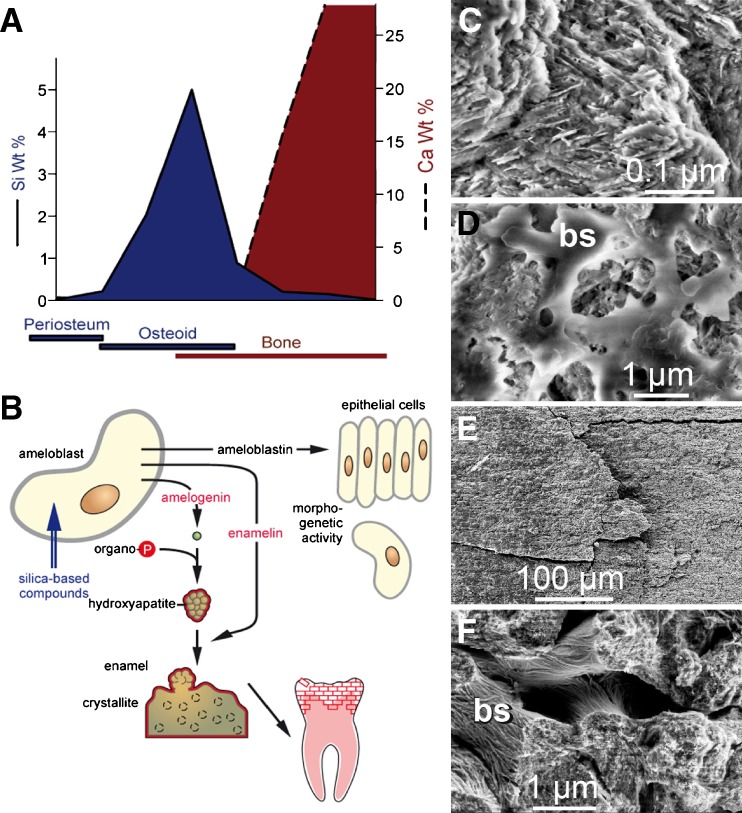
Biomedical application of biosilica and silicatein. **A** Spatial relationship between silicon accumulation and calcium composition during early stages of bone formation in rats (modified according to Carlisle 1986). **B** Schematic representation of the effect of silica-based components on the expression of the three marker genes (amelogenin, ameloblastin, enamelin) in ameloblasts. The silica-based components stimulate the expression of amelogenin, resulting in the formation of nanospherical hydroxyapatite around which hydroxyapatite crystals are deposited. **C**, **D** Formation of biosilica layers on pig molars. After tissue removal, the teeth were treated with phosphate buffered saline, supplemented with protease and phosphatase inhibitors, according to Aoba et al. (1987). Subsequently, dental hydroxyapatite was incubated with sodium metasilicate (100 μM) in the absence (**c**) or presence (**D**) of recombinant silicatein (4 μg/ml PBS) for 12 h at 20°C. Then, the samples were examined by HR-SEM. **E**, **F** In parallel, biosilica formation on femur bone samples was examined: untreated control (**E**) or silicatein-treated (**F**). The biosilica layers are marked (*bs*)

Biosilica is an essential nutrient for the natural ecosystem in general (Struyf and Conley 2008), for humans and other vertebrates in particular (Carlisle 1986; Dyck et al. 2000), where silicon deprivation results in severe skeletal malformations (Carlisle 1972). Thus, in avian connective tissue, the highest silicon concentrations were determined as compared to heart or muscle tissue. Moreover, a spatial correlation could be established between the areas of bone formation within animal tissue and the accumulation of silicon (Fig. 10A). Thus, a burst of silicon accumulation was seen around the osteoid and osteoid-bone interfaces, suggesting that this inorganic component is essential for bone formation. Consequently, the effect of biosilica on the activity of osteoblasts was investigated in depth. Indeed, the cell model used (human osteogenic sarcoma cells; SaOS-2) displayed an increased mineralization activity, when cultivated on biosilica surfaces in the presence of β-glycerophosphate, an organic phosphate donor (Schröder et al. 2005). In particular, concurrent coating of the substratum with biosilica and type 1 collagen not only increased the cellular Ca-phosphate deposition but also stimulated cell proliferation. In subsequent studies, the effect of biosilica and silica-based components on the expression of key genes involved in formation of tooth enamel—amelogenin, ameloblastin, and enamelin—was investigated. These studies revealed that the combination of β-glycerophosphate and silica-based components increased the expression of these marker genes (Fig. 10B). This finding was further supported by HR-SEM, visually demonstrating the increased deposition of hydroxyapatite crystallites in treated cells (Müller et al. 2007b).

First attempts to evaluate the biomedical application of silicatein/biosilica for treatment of bone/tooth defects and dental care are promising. Thus, during a reaction of silicatein with the substrate sodium metasilicate, nanoscale biosilica layers (50–150 nm) were formed on dental (pig teeth; Fig. 10C, D) and bone hydroxyapatite (rat femurs; Fig. 10E, F; HR-SEM). This data pave the way for biomedical approaches that aim, e.g., to generate protective biosilica layers on teeth (reducing the risk of bacterial induced caries/cavities) or to regenerate bone tissue via biosilica-stimulated activity of mineralizing cells (resulting in an increased deposition of hydroxyapatite).

### Biotechnological approach

**Fig. 11:**
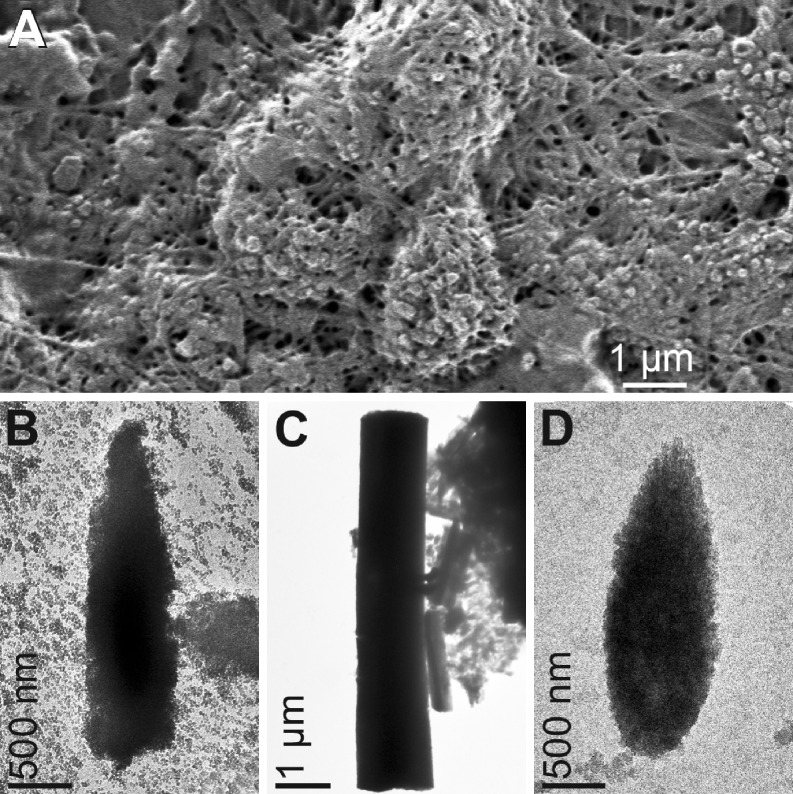
Synthetic axial filaments and spicules. **A** Formation of filaments through silicatein-a and silintaphin-1 interaction in vitro (TEM). **B** Assembly of silica nanoparticles in the presence of silicatein-α and silintaphin-1 to distinct rod-like/spicular shapes in vitro (SEM). **C** Silintaphin-1 interaction with silicatein-α immobilized on functionalized γ-Fe_2_O_3_ nanoparticles also directs formation of ordered structures. **D** Incubation of silicatein-α and silintaphin-1 with titanium *bis*-(ammonium-lactato)-dihydroxide resulted in the synthesis of nanostructured biotitania that assembled to spicular structures (SEM)

The discovery of silintaphin-1 established the basis to control in vitro biosilicification since during bioinspired approaches, coincubation of refolded recombinant silicatein and silintaphin-1 (equimolar concentrations) resulted in the formation of biomimetic filamentous protein structures with a diameter of 20–40 nm and a length of several micrometers, strongly resembling natural axial filaments. Figure 11A depicts a mesh of interwoven filaments consisting of both proteins. Reactions that excluded either silicatein or silintaphin-1 did not display such structures. These observations confirmed that silintaphin-1 represents a scaffold protein that is required for the filamentous organization of silicatein also in vivo.

In poriferan cells, spiculogenesis is initiated by the synthesis of the axial filament around which silica nanoparticles assemble to form a compact concentric layer. In a further biomimetic approach, this process was reconstructed in vitro. For that purpose, amorphous silica nanoparticles (diameter 20 nm) were coincubated with refolded recombinant silicatein (1 h, RT). Fourier transform infra-red spectroscopy with attenuated total reflectance (FT-IR ATR) and immunoblot analyses confirmed the adsorption of the enzyme onto the nanoparticles’ surfaces even after several washing steps, indicative of the significant binding capacity of silicatein to amorphous silica. Subsequent incubation of the silica/protein complexes with native silintaphin-1 resulted in the formation of structures with spicular to rod-like morphology (200–400 nm in width, >2 μm in length), thus revealing the cross-linking capacities of silintaphin-1 (Fig. 11B). This visual inspection by TEM was corroborated by FT-IR ATR and Western blot analyses that demonstrated a strong interaction of silintaphin-1 with adsorbed silicatein (Wiens et al., in preparation). In controls, lacking silintaphin-1, no assembly of nanoparticles was observed.

In a variation of this experiment, the silica nanoparticles were exchanged by γ-Fe_2_O_3_ nanocrystals (diameter 8–10 nm) that had been surface-functionalized with the reactive polymer poly(pentafluorophenyl acrylate) and chemisorbed Ni^2+^-NTA to facilitate binding of recombinant, His-tagged silicatein (Tahir et al. 2006; Wiens et al. 2009). Neither functionalized (carrying silicatein) nor unmodified did the particles assemble to regular structures. However, coincubation of functionalized silicatein-binding γ-Fe_2_O_3_ nanocrystals with silintaphin-1 resulted once more in the formation of rod-like structures with clear-cut edges (Fig. 11C).

During the aforementioned experimental variations, inorganic nanoparticulate carriers were employed to immobilize silicatein. In an alternative approach, nanoparticles were omitted. Instead, equimolar concentrations of silintaphin-1 and silicatein were coincubated for 30 min (RT) to induce protein–protein interaction. Then, the titania alkoxide precursor titanium bis-(ammonium-lactato)-dihydroxide was added (100 μM) for 1 h (RT). This monomeric alkoxide precursor was recently employed to generate nanostructured biotitania during a reaction that was mediated by surface-immobilized silicatein (Tahir et al. 2005). In this alternative approach, the combination of silicatein and silintaphin-1 not only induced the formation of titania nanoparticles but also resulted in their assembly to spicular structures, resembling those composed of silica or γ-Fe_2_O_3_ in size and shape (Fig. 11D). Consequently, silintaphin-1 forms the basis for in vitro synthesis of tailored materials since it facilitates (a) the generation of polymer products without prior surface immobilization of silicatein and (b) the assembly of nanoparticles (including enzymatic nanoparticulate products) to ordered structures.

Natural sponge spicules can act as optical glass fibers that transmit light with high efficiency (Müller et al 2006), as initially described by Cattaneo-Vietti et al. (1996). In addition, transmission of light is very selective since only wavelengths between 615 and 1,310 nm can pass through the spicules (sharp high- and low-pass filters). Spicules comprise a high refractive index core, a surrounding low-refractive-index cylindrical tube, and an outer portion with a progressively increasing refractive index. These optical characteristics of spicules are complemented by surprising mechanical properties based on the composite structure, lamellar architecture, and presence of dopants. Thus, spicules revealed enhanced fracture toughness (Sundar et al 2003; Aizenberg et al. 2004). Accordingly, it is tempting to employ the directed assembly of silica nanoparticles by the combined action of silicatein and silintaphin-1 to fabricate light-guiding micro- and nanostructured composites that represent an economical alternative to industrial glass fibers currently used in micro-optical approaches.
